# Patterns of Management of Positive Sentinel Lymph Nodes in Breast Cancer Patients After the American College of Surgeons Oncology Group Z0011 Trial: A Retrospective Study

**DOI:** 10.3390/cancers17223621

**Published:** 2025-11-11

**Authors:** Mohamad Hadi El Charif, Malak Ghezzawi, Nour Kalot, Joelle Allam, Rasha Kakati, Jaber Abbas, Hazem Assi, Eman Sbaity

**Affiliations:** 1 Department of Internal Medicine, American University of Beirut Medical Center, Beirut 1107 2020, Lebanon; me209@aub.edu.lb (M.H.E.C.); nkk06@mail.aub.edu (N.K.); ha157@aub.edu.lb (H.A.); 2 Faculty of Medicine, American University of Beirut, Beirut 1107 2020, Lebanon; mg96@aub.edu.lb (M.G.); jya03@mail.aub.edu (J.A.); rtk12@mail.aub.edu (R.K.); ja19@aub.edu.lb (J.A.); 3 Department of Surgery, American University of Beirut Medical Center, Beirut 1107 2020, Lebanon

**Keywords:** ACOSOG Z0011, early breast cancer, sentinel lymph node biopsy, axillary lymph node dissection, overall survival, disease-free survival

## Abstract

Background: Breast cancer is the most prevalent malignancy among women and the leading cause of cancer-related mortality. Sentinel lymph node biopsy (SLNB) has emerged as an alternative to axillary lymph node dissection (ALND), with the ACOSOG Z0011 trial demonstrating that SLNB alone yields equivalent oncologic outcomes to ALND for patients with 1–2 positive sentinel lymph nodes (SLNs). Objective: This study evaluates the adoption of Z0011 recommendations at the American University of Beirut Medical Center (AUBMC) and their impact on oncologic outcomes. Methods: This retrospective cohort study includes female patients with early-stage breast cancer (T1–T2, N0, M0) who underwent breast-conserving surgery with SLNB at AUBMC, between January 2011 and December 2016. Data were extracted from the institutional breast cancer database, including demographics, tumor characteristics, surgical management, and follow-up outcomes. Results: SLNB alone was adopted, following publication of the Z0011 trial, in 93.4% of patients, with 6.6% undergoing ALND. Among patients with 1–2 positive SLNs, 27.1% underwent completion ALND. The median number of lymph nodes retrieved increased from two to four over the study period. The most common molecular subtypes observed were luminal A (54.1%), characterized by a predominant stage T1 presentation and small tumor sizes (median tumor size, 1.4 cm). Among patients with 1–2 positive SLNs who underwent ALND, no recurrences or metastatic events were reported. The 5-year overall survival (OS) and disease-free survival (DFS) rates were 95.9% and 93.4%, respectively. No notable differences in OS and DFS were observed between the SLNB alone and ALND groups. Conclusions: Our study demonstrates that SLNB alone provides comparable oncologic outcomes to ALND, supporting its safety and effectiveness as an alternative for early-stage breast cancer patients with 1–2 positive SLNs. These findings extend the Z0011 trial’s applicability to diverse populations, reinforcing SLNB as a less invasive, safe alternative to ALND.

## Simple Summary

The Z0011 trial, published nearly 14 years ago, has become part of the standard of care for breast cancer patients worldwide. However, practices in Lebanon and other LMICs often do not align with international guidelines, potentially jeopardizing patient outcomes. This study demonstrates that at AUBMC, a tertiary referral center in the MENA region, physicians adhere to global recommendations and achieve positive oncologic results. Our findings show that ALND offers no survival advantage over SLNB alone in overall and disease-free survival, supporting the Z0011 conclusions and extending their applicability to an ethnically diverse population. Moreover, this report highlights the high quality of care and research at our academic medical center, despite resource limitations. Such capabilities allow us to serve patients in need of advanced breast cancer care who might otherwise seek treatment abroad.

## 1. Introduction

### Global Impact of Breast Cancer

Breast cancer continues to pose a significant global health challenge, ranking as the most common malignancy among women and the leading cause of cancer-related mortality [1,2]. According to the World Health Organization’s Global Cancer Observatory, in 2022 alone, there were approximately 2.3 million new cases and 666,000 deaths due to breast cancer worldwide [2]. These outcomes persist despite notable improvements in survival outcomes, achieved through advancements in early detection, systemic therapies, and surgical techniques [3,4].

Historically, axillary lymph node dissection (ALND) was the cornerstone of surgical management for breast cancer patients with clinically suspicious axillae or confirmed nodal involvement [5]. The procedure, which involves dissecting the axilla and removing multiple lymph nodes, served as both a staging tool and a therapeutic intervention aimed at reducing locoregional recurrence and improving survival [5]. However, ALND is associated with substantial morbidity, including lymphedema, shoulder dysfunction, and neuropathic pain, which can profoundly impact patients’ quality of life [5].

In recent decades, sentinel lymph node biopsy (SLNB) has revolutionized axillary management in clinically node-negative breast cancer patients, becoming the standard of care [6]. SLNB is a minimally invasive procedure that identifies and removes sentinel lymph nodes (SLNs) using tracers, such as Technetium-99m sulfur colloid or isosulfan blue, to detect potential metastatic spread [7]. SLNB has demonstrated comparable oncologic outcomes to ALND in terms of staging and survival, with significantly reduced post-operative morbidity [8]. Currently, ALND is reserved for patients with pathologically involved lymph nodes after neoadjuvant chemotherapy or those with extensive nodal involvement [9].

The American College of Surgeons Oncology Group (ACOSOG) Z0011 trial was a landmark study that challenged the traditional reliance on ALND for patients with positive SLNs [10]. The trial enrolled patients with early-stage breast cancer (clinical T1–T2, N0, M0) who had 1 or 2 positive SLNs and compared outcomes between those undergoing ALND and those managed with SLNB alone [10]. All patients underwent breast conservation therapy (BCT), whole-breast irradiation, and systemic therapy, depending on physician judgment [11,12]. The results, published initially in 2010–2011 and further reinforced in 2017, demonstrated no significant difference in disease-free survival (DFS) and overall survival (OS) between the two groups. However, SLNB alone markedly reduced surgical morbidity [5,10,13,14,15,16].

The ACOSOG Z0011 trial has fundamentally reshaped clinical practice, leading major oncology organizations, such as the National Comprehensive Cancer Network (NCCN), to update guidelines to recommend SLNB alone for selected patients with limited nodal involvement [17,18]. This shift represents a major paradigm change in breast cancer surgery, as institutions worldwide have adopted less invasive axillary management approaches, significantly reducing the use of ALND and sparing patients its associated morbidities [19,20,21]. One example of a trial supporting applicability across diverse populations is a study conducted on a Belgian cohort by Pop et al., which demonstrated that omitting ALND in Z0011-eligible patients did not increase the risk of disease recurrence. This suggests that SLNB alone may be a safe and effective approach in European populations as well [22].

Despite the widespread adoption of the Z0011 criteria, some controversies remain. Critics have highlighted the trial’s limitations, including its predominantly older patient cohort and relatively low nodal tumor burden, which may not fully represent the broader population of early-stage breast cancer patients [12]. Furthermore, its generalizability to various ethnic populations and diverse clinical settings, particularly in non-Western countries, remains an area of ongoing investigation.

This study aims to evaluate the adoption and outcomes of the Z0011 trial’s findings in an eligible cohort of breast cancer patients treated at the American University of Beirut Medical Center (AUBMC) during the first five years following the trial’s publication. The findings are expected to demonstrate the applicability of Z0011 recommendations to populations with diverse ethnic and genetic backgrounds, as well as reflect evolving clinical practices at centers in low- to middle-income countries (LMICs).

## 2. Methods

### 2.1. Study Design and Population

This retrospective epidemiologic cohort study was conducted at the American University of Beirut Medical Center using data from an institutional breast cancer database curated by the Medical Archives Department. The study includes female patients diagnosed with early-stage breast cancer (cT1-T2N0M0) and treated between January 2011 and December 2016. Eligible patients were those who underwent upfront breast-conserving surgery (BCS) with SLNB, with or without subsequent ALND. Patients who received neoadjuvant chemotherapy or presented with distant metastases at diagnosis were excluded. Ethical approval for the study was obtained from the Institutional Review Board at AUBMC under IRB ID # BIO-2019-0419, with a waiver of informed consent granted due to the retrospective nature of the study.

### 2.2. Data Collection

Comprehensive data were extracted, including demographic information such as age and gender, as well as medical and family history, particularly any relevant comorbidities or history of breast or other cancers. Clinical presentation details were collected, including the presence of palpable masses or axillary nodes, along with radiological evaluations (ultrasound, mammography, MRI, and PET-CT) to assess initial tumor characteristics. Tumor-specific information, such as biopsy pathology, immunohistochemistry, and both clinical and pathological staging, along with details of surgical management, including SLNB and ALND procedures, were documented. Information on adjuvant therapies received by patients, including chemotherapy, hormonal therapy, and radiotherapy, is also recorded. Follow-up data were collected regarding disease recurrence (local, distant, or both) and survival outcomes to calculate DFS and OS.

### 2.3. Definitions

Patients were categorized into molecular subtypes based on immunohistochemical analysis following widely recognized criteria [23,24,25]. Tumors were classified as luminal A (ER and/or PR positive, HER2 negative), luminal B (ER and PR positive, HER2 positive or negative), HER2-enriched (ER and PR negative, HER2 positive), or triple-negative (ER, PR, and HER2 negative). SLNB procedures at AUBMC were performed using one or both of the following techniques: intraoperative injection of patent blue dye into the breast parenchyma or injection of radioactive colloid detectable with a gamma probe. Nodes identified as radioactive and/or blue-stained were excised and submitted to the pathology laboratory for examination, including frozen section analysis when required.

### 2.4. Study Endpoints

The primary endpoint of this study is to assess the concordance of AUBMC’s management of early breast cancer patients—specifically regarding the omission of ALND in cases with T1/T2 tumors and 1–2 SLNs—with the recommendations established by the Z0011 trial, and to compare our institutional outcomes to those reported in Z0011. Secondary endpoints included the entire cohort’s DFS and OS.

### 2.5. Statistical Analysis

Descriptive statistical analysis was used to summarize demographic, clinical, and treatment variables. Stratified descriptive analysis was also applied to describe characteristics of specified subgroups. Kaplan–Meier survival analyses were used to estimate DFS and OS. All statistical analyses were conducted using IBM SPSS version 20.0.

## 3. Results

### 3.1. Patient Demographics

**Figure 1:**
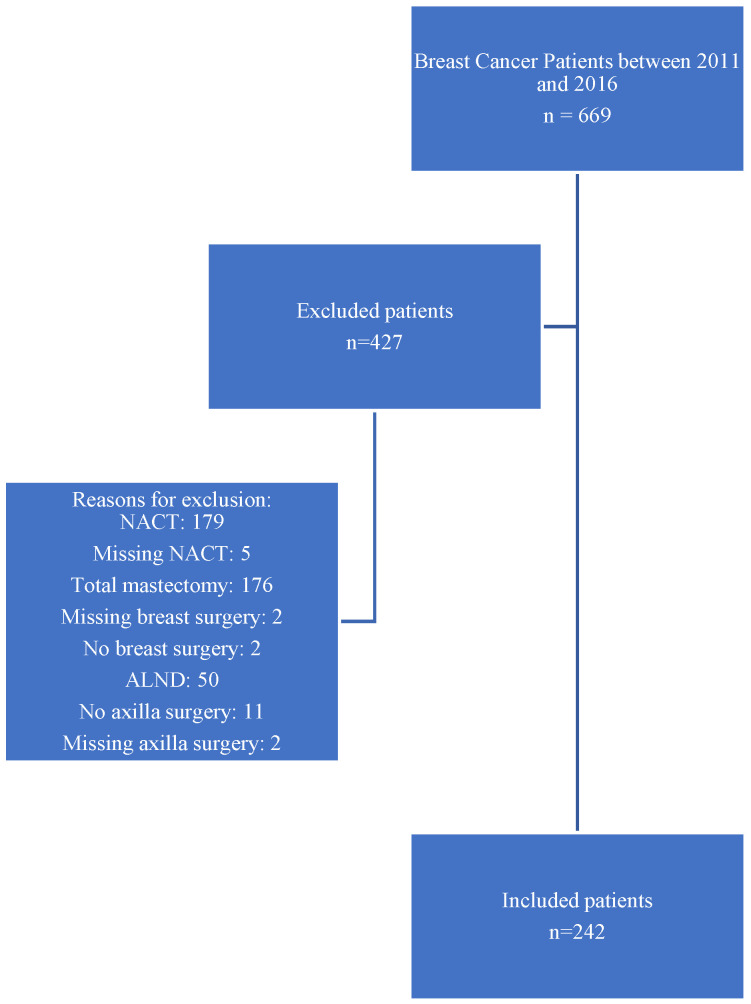
Flowchart of patient selection for inclusion in the Z0011-eligible early breast cancer cohort at AUBMC (2011–2016).

A total of 669 patients with early breast cancer were screened for inclusion, of whom 242 cases were included and 427 excluded for the following reasons: 179 had received NACT; 176 underwent total mastectomy; 50 underwent ALND without sentinel biopsy; 11 did not undergo axillary surgery; two did not undergo breast surgery; nine patients had incomplete or missing data regarding NACT, breast surgery, or axillary surgery. These numbers are summarized visually in the flowchart presented in Figure 1.

Thus, the study cohort comprised 242 female patients with biopsy-proven, primary, non-metastatic breast cancer treated at AUBMC between January 2011 and December 2016. This cohort included six patients with bilateral breast cancers. The median age of the patients was 52.8 years (range: 28.6–83.6). Patient characteristics are further described in Table 1.

### 3.2. Tumor Characteristics and Lymph Nodes Status

The median tumor diameter was 1.4 cm (range: 0.13–5.0 cm). The most common type of breast cancer in this cohort was invasive ductal carcinoma (IDC), occurring in 85.1% (206/242) of patients. Most patients (193/242, or 80.5%) presented with T1 tumors. Regarding molecular subtypes, most patients (131/242, 54.1%) were classified as Luminal A, while only 3.3% (8/242) had HER2-positive tumors. Tumor grade distribution was as follows: 64 patients (26.6%) had grade 1 tumors, 110 patients (45.6%) had grade 2 tumors, and 67 patients (27.8%) had grade 3 tumors. Further details on tumor characteristics are provided in Table 1.

### 3.3. Surgical Management

All patients underwent upfront BCS with ALN management. Of the 242 cases, 222 (91.7%) underwent SLNB alone, while 20 (8.3%) underwent ALND. Lymphatic mapping was performed using radioactive colloid alone in 27.3% of cases, blue dye alone in 31.8%, and a combination of both in 40.9% of cases. The median number of lymph nodes retrieved during the first SLNB procedure was 3 (range 1–12).

### 3.4. Adjuvant Therapies

Among patients with available data on adjuvant therapy, 61.1% (143/234) received systemic therapy, 87.7% (207/236) received radiation therapy, and 60.4% (136/225) received hormonal therapy.

### 3.5. Changes in ALN Management Post-Z0011

Axillary management at AUBMC was evaluated for the period between January 2011 and December 2016, covering a total of 242 patients. During this period, the proportion of patients who underwent SLNB alone was 93.4% (222/242), while 6.6% (20/242) underwent ALND.

Overall, 78.6% (190/242) of patients had negative SLNs, and of these, 2.1% (4/190) proceeded to ALND. In contrast, 21.5% (52/242) of patients had positive SLNs, of which 30.8% (16/52) underwent completion ALND. Specifically, 19.8% (48/242) of patients had 1–2 positive SLNs, and 27.1% (13/48) of those underwent ALND.

Following publication of Z0011, practice patterns shifted. Between 2011 and 2013, 18.5% (15/81) of patients had 1–2 positive nodes, of which 46.7% (7/15) underwent completion ALND. From 2014 to 2016, 20.5% (33/161) of patients had 1–2 positive nodes, but only 18.2% (6/33) underwent ALND. This reflects a decline in completion ALND from nearly 1 in 2 patients early in the post-Z0011 period to roughly 1 in 4 in the later years, following the publication of long-term survival data.

### 3.6. Subgroup Descriptive Analysis

This Section describes tumor and axillary characteristics for the 13 patients who underwent ALND out of the 48 patients with 1 or 2 positive SLNs. The median age at diagnosis for this subgroup was 52.9 years (range: 36–67). Most were diagnosed with T1 tumors (9, 69.2%), and the median pre-operative tumor size was 1.6 cm (range: 0.7–3.5). The majority had grade 2 tumors (8, 61.5%) and luminal A subtype (7, 53.8%).

Regarding axillary involvement, the median number of sentinel lymph nodes was 2 (range: 1–9). Ten out of the 13 patients (76.9%) had one positive SLN, while three (23.1%) had two positive SLNs. The median size of tumor deposits in retrieved SLNs was 10 mm (range: 2–28), indicating macro-metastasis in all cases. Only 15.4% (2/13) of patients had additional positive nodes found on completion of ALND.

None of the patients experienced recurrence or metastatic events during a median follow-up of 62.4 months (range: 1–104 months). Detailed tumor characteristics for these patients are presented in Table 2.

### 3.7. Oncologic Outcomes

Follow-up data were available for 240 patients, with a median follow-up period of 50 months (range: 1–100 months). A total of 11 patients (4.6%) experienced 13 events of disease recurrence, as summarized in Table 3. These included three cases with locoregional recurrence (LRR), seven cases with distant recurrence (DR), and one case of both LRR (local and nodal) and DR. The recurrence rate in the SLNB followed by ALND group was 5% (1/20); this event was a distant recurrence event. All patients underwent SLNB, except for one case in the DR group, who also underwent ALND.

**Figure 2:**
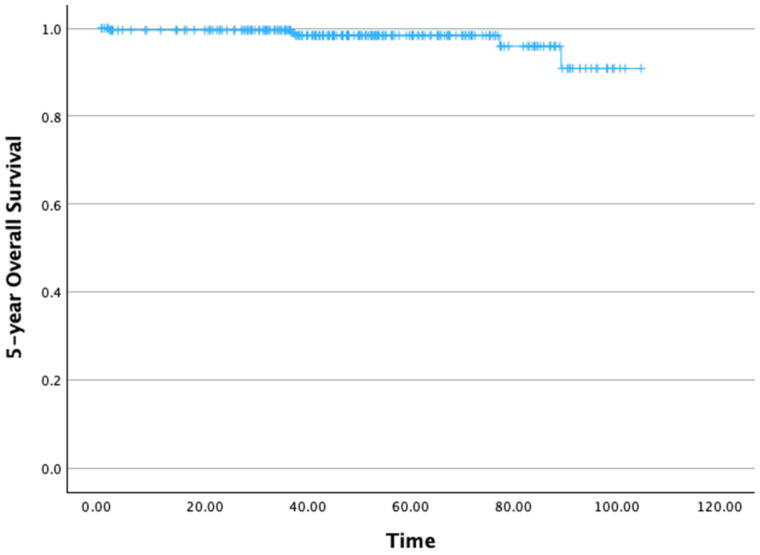
Five-year overall survival.

The 5-year OS rate was 95.9% (Figure 2). The mean 5-year OS time was 100.96 months (standard deviation [SD]: 1.22 months), with a 95% confidence interval (CI) ranging from 99.56 to 104.36 months.

**Figure 3:**
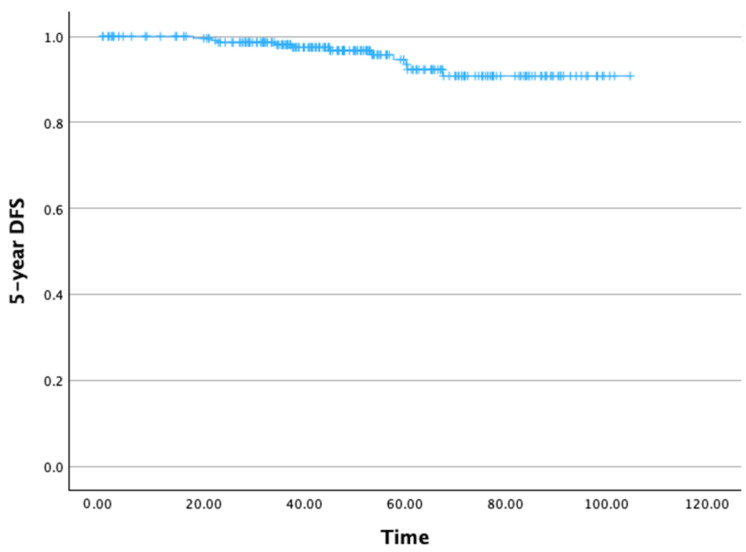
Five-year disease-free survival.

The 5-year DFS rate was 93.4% (Figure 3). The mean 5-year DFS time was 99.65 months (SD: 1.47 months), with a 95% CI ranging from 99.77 to 102.53 months.

## 4. Discussion

### 4.1. Patient Demographics and Tumor Characteristics Comparability

The demographic and tumor characteristics of our cohort were comparable to those in the Z0011 trial. The median age in our cohort closely aligns with the 55-year-old median in the Z0011 cohort. Similarly, the median pre-operative tumor size in our study aligns with the trial’s findings of 1.6 cm and 1.7 cm (range 0–5 cm and 0.4–7 cm for the SLNB alone and ALND arms, respectively). Histologically, invasive ductal carcinoma (IDC) was the predominant subtype in both our cohort and the Z0011 trial, comprising 85.1% of cases in our study, compared to 81.7% and 81.9% in the trial arms, respectively. Invasive lobular carcinoma (ILC) accounted for 10.3% of our cases, compared to 8.3% and 6.4% in the Z0011 arms [10].

Hormone receptor (HR) status analysis showed that most cases in our cohort were ER+/PR+ (162/242), followed by ER−/PR− (28/242), ER+/PR− (18/242), and ER−/PR+ (5/242). This distribution differs somewhat from Giuliano et al., who reported more ER+/PR− cases and fewer ER−/PR− cases. However, both cohorts showed a predominance of ER+/PR+ status [14]. A large German cohort study of 13,741 Z0011-eligible patients reported 90.7% ER+, 81.4% PR+, and 90.1% HER2-negative tumors [26], suggesting regional variability in HR expression. The higher ER−/PR− proportion in our cohort may reflect underlying ethnic or genetic differences and underscores the need for further molecular studies in diverse populations.

Grade 2 tumors were the most common in our cohort, followed by grades 1 and 3—a distribution like that in the Z0011 trial [14]. Jung et al. also reported a predominance of grade 2 tumors (56.8%) in their large Asian cohort, supporting the consistency of this pattern [27]. Tumor focality was predominantly unifocal in our cohort, in contrast to Chung et al., who found multifocality in 23% of their Z0011-eligible cohort [28]. These differences may stem from population variability or differences in imaging and pathological assessment methods.

### 4.2. Nodal Disease Status and Surgical Implications

In our study, clinically node-negative (cN0) patients generally had negative SLNs on final pathology, consistent with the findings from Kim, Giuliano, and Lyman [29]. However, the incidence of micro-metastasis in our study was lower than the reported rates in the literature (range 25–46%) [30], possibly due to our consistent use of preoperative axillary ultrasound for more accurate staging of ALNs. Conversely, the incidence of macro-metastasis in our cohort was higher than the 50–55% seen in studies from the Western cohort [31,32,33]. These variations may reflect differences in patient populations, local practices, or the methods used to assess nodal status. Data on ALN status and methods of evaluation are reported in Supplementary File S1.

Interestingly, in cases with more than two positive SLNs, the Z0011 trial opted for completion of ALND [26]. For those with SLN positivity, whole-breast radiation, with or without adjuvant chemotherapy, is the recommended approach [34]. More recent studies suggest that even in cases of macro-metastasis, ALND may be safely omitted in favor of radiation and/or systemic therapy [35,36]. Kuru et al. reported favorable outcomes using this approach with minimal rates of axillary recurrence, lymphedema, and shoulder restriction [36]. Despite these evolving strategies, Z0011 remains a key reference for axillary management in early breast cancer, particularly in cases with macroscopic nodal disease [35,37]. This is particularly significant given the high prevalence of macro-metastasis in its cohort, compared with the modest 35% micro-metastasis rate in early breast cancer patients [35,37]. Our findings support this approach, with favorable oncological outcomes even in patients with macro-metastasis.

### 4.3. Surgical Nodes and Personalized Medicine

The observed trend of increasing SLNB rates and decreasing ALND use in our cohort mirrors global practice patterns following the Z011 publication [5]. This shift reflects both a move toward conservative axillary management and the broader adoption of personalized treatment approaches in early breast cancer. The preference for SLNB over ALND is evident not only in Europe but also in parts of the Middle East and North Africa (MENA) region [16,26].

The median number of sentinel nodes retrieved in our study was lower compared to the Z0011 trial. Giuliano et al. report a median of 17 nodes with an interquartile range (IQR) of 13–22 in the ALND arm [11]. In contrast, Jung et al. reported that 44.7% of the Asian cohort had three or more nodes retrieved, while the remaining cases had two or fewer nodes retrieved [27]. SLNs status, number retrieved, and method of detection are reported in Supplementary File S2.

### 4.4. Adjuvant Treatment and Radiotherapy

Most patients in our cohort received systemic therapy and radiation therapy, and a considerable proportion received hormonal therapy. In comparison, Giuliano et al. had even higher rates of adjuvant therapy (96–97%) in the Z0011 trial arms [14]. Regarding radiation therapy, both our cohort and the Z0011 trial show similar adherence, with a slightly higher percentage of patients in the Z0011 trial receiving it (88.9% and 89.6% in the respective arms), compared to our cohort [14]. Despite the differences in proportions, both studies emphasize the central role of systemic therapy and radiotherapy in the management of axillary metastasis. Thus, highlighting the critical role of these treatments in adjuvant care, with the trends underscoring their importance in axillary metastasis management.

### 4.5. Oncological Outcomes Comparability

The oncological outcomes observed in our cohort are consistent with those reported in the Z0011 trial, supporting the applicability of its findings to diverse populations. The Z0011 trial reports a 5-year OS of 92.5% and a 5-year DFS of 83.9% for the SLNB-alone group [11]. These findings suggest that SLNB alone is a viable treatment for early breast cancer with clinically node-negative axillae cN0. This strengthens the generalizability of the Z0011 findings, particularly to populations in Europe, Eastern/Western Asia, and the MENA region [38]. Genomic studies from the Levant Region suggest significant genetic similarities between Mediterranean populations and nearby Asian, European, and African regions [38], which may further support the applicability of these findings across diverse populations.

Furthermore, the low rates of LRR in our cohort are consistent with those observed in other studies, including Giuliano et al. (3.4% LRR) [10]. Furthermore, the incidence of distant metastasis and the median time to distant recurrence in our cohort align with findings by Jung et al., who report a 3.8% distant recurrence rate [27]. These comparable outcomes highlight the long-term efficacy of SLNB and adjuvant treatments in early breast cancer management.

### 4.6. AUBMC: Conforming with International Guidelines

At our institution, the practice of breast cancer surgery has evolved in congruence with international guidelines that incorporated the Z0011-based recommendations. For instance, we found that the rate of ALND decreased markedly over time, reflecting the shift toward SLNB alone in the management of early breast cancer with cN0. A subset of 13 patients, with two or fewer positive nodes and who underwent ALND, had nodes with large tumor deposits, and most of these patients had a family history of breast cancer. These factors may have influenced the surgeon’s decision to proceed with completion ALND, considering the potential confounding effects of family history and larger nodal deposits, which have not been extensively studied.

### 4.7. The Global Impact of the ACOSOG Z0011 Trial

The ACOSOG Z0011 trial has shaped breast cancer management guidelines worldwide, impacting practices for the treatment of early-stage breast cancer over the past decade [15]. This is evident in a study by Caudle et al. at MD Anderson Cancer Center, which included 658 patients, and found that the rate of ALND decreased from 85% to 24% (*p* < 0.001) in patients with positive SLNs after the publication of Z0011 in 2011 [39]. Similarly, a Canadian multicenter study of 3291 cases by Tsao et al. found a decline in the rate of ALND from 71% to 17% after the Z0011 recommendations were implemented [40]. Moreover, another report from Pakistan revealed that the rate of ALND decreased markedly from 100% to 17% when comparing patients presenting before and after the introduction of Z0011-based recommendations [21].

Several international studies have further validated the applicability of the Z0011 results in diverse populations. For instance, one study by Jung et al. reviewed the medical records of 1750 patients and found that the Z0011-based approach to managing early breast cancer with cN0 is safe and effective for Asian populations (adj. HR after ALND omission is 0.83 (95% CI 0.34–2.03) among patients with ≤2 SLNs) [27].

In our original breast cancer database, which includes cases from 2011 to 2016 and excludes patients with metastatic breast cancer, we found that 29% of cases (242/810) were eligible for the Z0011-based recommendations. This proportion is consistent with findings from other studies, where the percentage of breast cancer patients who meet Z0011 eligibility criteria can reach up to 22% [41,42]. These figures highlight the substantial proportion of the breast cancer population that would be affected by adopting the Z0011 approach to managing ALNs.

## 5. Conclusions

Our study, with a follow-up period extending more than five years, demonstrates that SLNB alone offers comparable rates of OS and DFS to ALND, while avoiding associated morbidities. Although the ACOSOG Z0011 trial was published over a decade ago, supporting the omission of ALND in a broad group of SLNB-positive patients, ALND is still commonly performed in cases with fewer than two positive SLNs.

At our institution, surgeons rapidly adopted the Z0011-based guidelines, reflecting their relevance and applicability. Our findings not only conform to the ACOSOG Z0011-based recommendations but also contribute to their generalizability to diverse populations worldwide, reinforcing the potential of SLNB as a safe and effective option for early-stage breast cancer management.

## Figures and Tables

**Table 1:** Patient and tumor characteristics in a cohort of Z0011-eligible patients at AUBMC.

Characteristics		Count (N = 242) (%)	Percentage
Patient Characteristics			
Age at Diagnosis (years)	Median (Range)	52.79 (28.56–83.62)	
Family History of Cancer	Yes	121	51.5%
	No	114	48.5%
Family History of Breast Cancer	Yes	84	37.8%
	No	138	62.2%
Personal History of Cancer	Yes	9	3.7%
	No	233	96.3%
**Tumor Characteristics**			
Pre-Operative Tumor Size (cm)	Median (Range)	1.4 (0.13–5)	
Grade	1	64	26.6
	2	110	45.6
	3	67	27.8
Type	IDC	206	85.1
	ILC	25	10.3
	IDC and ILC	4	1.7
	Others	7	2.9
Subtype	Luminal A	131	54.1
	Luminal B	82	33.9
	HER2	8	3.3
	Triple Negative	21	8.7
Focality	Yes	33	13.6
	No	209	86.4

IDC: Invasive Ductal Carcinoma; ILC: Invasive Lobular Carcinoma.

**Table 2:** Tumor characteristics in cases with two or fewer positive SLNs that underwent ALND.

Cases with 1 or 2 Positive SLNs + ALND		Count (N = 13) (%)
**Pre-Op Tumor Size (cm)**	<1 cm	1 (7.7)
	1–2 cm	9 (69.2)
	>2 cm	3 (23.1)
Grade	1	2 (15.4)
	2	8 (61.5)
	3	3 (23.1)
Type	IDC	11 (84.6)
	ILC	2 (15.4)
	IDC and ILC	0 (0.0)
	Others	0 (0.0)
Subtype	Luminal A	7 (53.8)
	Luminal B	2 (15.4)
	HER2	2 (15.4)
	Triple Negative	2 (15.4)
Number of SLNs Retrieved (median: 3; range: 1–12)	1 or 2	7 (53.8)
	3 or 4	4 (30.8)
	5 or More	2 (15.4)

IDC: Invasive Ductal Carcinoma; ILC: Invasive Lobular Carcinoma.

**Table 3:** Recurrence event rates.

Event	Count (N = 240) (%)	ALND Group
LRR	4 (1.7)	0/4
NR	1 (0.4)	0/1
DR	8 (3.3)	1/8

## Data Availability

The datasets generated and/or analyzed during the current study are not publicly available due to institutional policies, but are available from the corresponding author upon reasonable request.

## Supplementary Materials

The following supporting information can be downloaded at: https://www.mdpi.com/article/10.3390/cancers17223621/s1, Supplementary File S1: Axillary Lymph Node Status Stratified by Investigational Modality. Supplementary File S2. Surgical Sentinel Lymph Nodes Detection & Retrieval.

## Abbreviations

ACOSOG	American College of Surgeons Oncology Group
SLN	Sentinel Lymph Node
SLNB	Sentinel Lymph Node Biopsy
ALND	Axillary Lymph Node Dissection
LN	Lymph Node
NSLN	Non-SLN
cN0	Clinically Node Negative
BCT	Breast-Conservation Therapy
DFS	Disease-Free Survival
NCCN	National Comprehensive Cancer Network
U.S.	United States
AUBMC	American University of Beirut Medical Center
BCS	Breast-Conserving Surgery
IRB	Institutional Review Board
OS	Overall Survival
IDC	Invasive Ductal Carcinoma
ILC	Intralobular Carcinoma
HER2	Human Epidermal Growth Factor Receptor 2
ALN	Axillary Lymph Node
LRR	Loco-Regional Recurrence
DR	Distant Recurrence
MRI	Magnetic Resonance Imaging
IQR	Interquartile Range
MENA	Middle East and North Africa
Adj. HR	Adjusted Hazards Ratio
