# Supplementary material for: Patterns of Management of Positive Sentinel Lymph Nodes in Breast Cancer Patients After the American College of Surgeons Oncology Group Z0011 Trial: A Retrospective Study
Source: Cancers (Basel). 2025 Nov 11;17(22):3621. doi: 10.3390/cancers17223621 (PMC12650466; doi:10.3390/cancers17223621)
Supplement: Supplementary file 1 [file cancers-17-03621-s001.zip › cancers-3724597-supplementary.pdf]

Supplementary Material:

Supplementary File S1. Axillary Lymph Node Status Stratified by Investigational Modality

ALN Status by Investigational Modality		Count (N=242)(%)
**ALN Status on PET**	Positive	10 (4.1)
	Negative	80 (33.1)
	Not Done	152 (62.8)
**ALN Status on US**	Positive	25 (10.3)
	Negative	198 (81.8)
	Missing	19 (7.9)
**ALN Status on FNA**	Negative	22 (9.1)
	Positive	5 (2.1)
	Inconclusive, Suspicious or Atypical	4 (1.7)
	Not Done	211(87.2)

Supplementary File S2. Surgical Sentinel Lymph Nodes Detection & Retrieval

Surgical SLN Specimen		Count (N=242)(%)
**Method of Sentinel Lymph Node Detection**	Blue Dye	77 (31.8)
	Radioactive Colloid	66 (27.3)
	Both	99 (40.9)
**Number of SLNs Retrieved (median: 3; range: 1-12)**	2 or Less	95 (39.4)
	3 or 4	90 (37.4)
	5 or More	56 (23.2)
**Number of Positive SLNs Retrieved**	None	190 (78.5)
	1 or 2	48 (19.8)
	More than 2	4 (1.7)
